# A novel arylbenzofuran induces cervical cancer cell apoptosis and G1/S arrest through ERK-mediated Cdk2/cyclin-A signaling pathway

**DOI:** 10.18632/oncotarget.9731

**Published:** 2016-05-31

**Authors:** Pinghong Ming, Ting Cai, Jin Li, Yong Ning, Shengao Xie, Tao Tao, Faqing Tang

**Affiliations:** ^1^ Department of Clinical Laboratory and Medical Research Center, Zhuhai Hopital of Jinan University, Zhuhai People's Hospital, Zhuhai, 519000, China; ^2^ Hubei University of Traditional Chinese Medicine, Wuhan, 430065, China; ^3^ Shenzhen Second People's Hospital, Shenzhen, 518055, China; ^4^ China State Institute of Pharmaceutical Industry, Shanghai, 200437, China

**Keywords:** cervical cancer, arylbenzofuran, apoptosis, mitochondria, cell cycle

## Abstract

7-hydroxy-5,4′-dimethoxy-2-arylbenzofuran (Ary) is purified from Livistona. It has been demonstrated to have anticancer activity to various tumors in including cervical cancer, but its mechanism is still unclear. In the present, we show that Ary induces cervical cancer cells apoptosis through mitochondria degradation and mediates cervical cancer cell arrest. Further, Ary-inducing cell cycle G1/S-phase arrest is associated with increased cyclin A2 and cyclin dependent kinase 2 (Cdk2) proteins. Knockdown of cyclin A2 using small interfering RNA (siRNA), and inhibiting Cdk2 activity with flavopiridol, strikingly reduced G1/S-phase arrest. Moreover, Ary sustainedly induced phosphorylation of extracellular signal-regulated kinase1/2 (ERK1/2). And ERK1/2 phosphorylation inhibition using specific inhibitor U0126 effectively suppressed cyclin A2 expression, and reduced G1/S-phase arrest induced by Ary. All the experiments *in vitro* and *in vivo* verified that Ary has an anticancer effect on cervical cancer. These data provide novel evidences that Ary induces cervical cancer cells apoptosis through mitochondria degradation and cell G1/S-phase arrest. These findings also suggest that ERK-mediated Cdk2/cyclin A signaling pathway is involved in Ary-induced G1/S-phase arrest.

## INTRODUCTION

Cervical cancer is a major common malignant tumour of women in worldwide, which is next only to breast cancer and colorectal cancer in developing countries [1, 2]. Many risk factors relate to cervical cancer pathogenesis, including viral infection, genetic mutation, telomerase activation, hormone abnormality, malnutrition, and immune dysfunction. In viral investigations, especially based on human papillomavirus (HPV), epidemiological studies showed that 99.4% invasions of cervical cancer patients are infected with HPV virus [3]. High risk-HPV (HR-HPV) infection is considered to be a major risk factor of cervical cancer development and precancerous lesions [4–6], such as HPV16, 18, 31, and 33 promote in cervical cancer pathogenesis [7–9]. Among all these types of HPV, types 16 and 18 are the most dangerous factors which are approximately 70 percent in cervical cancer [10, 11], but vaccine against HPV could not effectively prevented pre-existing HPV infections and progression of HPV-associated lesions. In current, the therapeutic regimens of cervical cancer include surgical removal, radiotherapy and chemotherapy. Women with early-stage cervical cancer can be potentially cured with radical hysterectomy or chemoradiation therapy [12]. Unfortunately, up to 17% of women develop either local and/or distant disease recurrence usually within the first 2 years of completing the treatment [13]. Local recurrence of cervical cancer after primary surgical therapy is also problematic, treatment directed to the site of recurrence can be performed with curative intent. Options include radiation therapy (RT) and pelvic exenteration, both resulting in suboptimal rates of local tumor control and rates of survival [14]. Chemotherapy is an important therapeutic tool, especially for the patients in advanced and metastatic. However, in the course of chemotherapy, some cancer cells may have varying degrees of drug resistance phenomenon. Most of chemotherapy drug toxicities are large, and the patients have a lot of adverse reactions after chemotherapy. Therefore, to search for a novel effective and harmless chemotherapy drug for cervical cancer is very necessary.

Livistona, a perennial tropical and subtropical evergreen tree, is mainly distributed in southern or south-eastern of China. Its seeds have been used for therapeutic esophageal cancer, nasopharyngeal carcinoma, malignant mole and leukemia [15–18]. Livistona chinensis fruit has been used for anti-hyperlipidemic and anti-ulcer [19]. 7-hydroxy-5,4′-dimethoxy-2- arylbenzofuran (Ary) is purified from *Livistona*, it has been found to inhibit the proliferation of tumor cells including HL-60, Mata, HepG2 and CNE-1 cells [20]. Our previous works showed that Ary (also named as HDAB) activates ATM-dependent DNA repair response and suppresses PARP-1 activity through docking into PARP-1 hydrophobic pocket, resulting in cell cycle arrest [21]. In the present, we showed that Ary can inhibit cervical cancer cell proliferation through G1/S arrest and initiate cervical cancer cells programmed to death by mitochondrial apoptosis way, therefore activate extracellular signal-regulated kinase (ERK) phosphorylation to elicit cell G1/S phase arrest through cyclin A2 and cyclin dependent kinase (Cdk) 2 upregulation.

## RESULTS

### Inhibitory effect of Ary on cervical cancer cell

**Figure 1:**
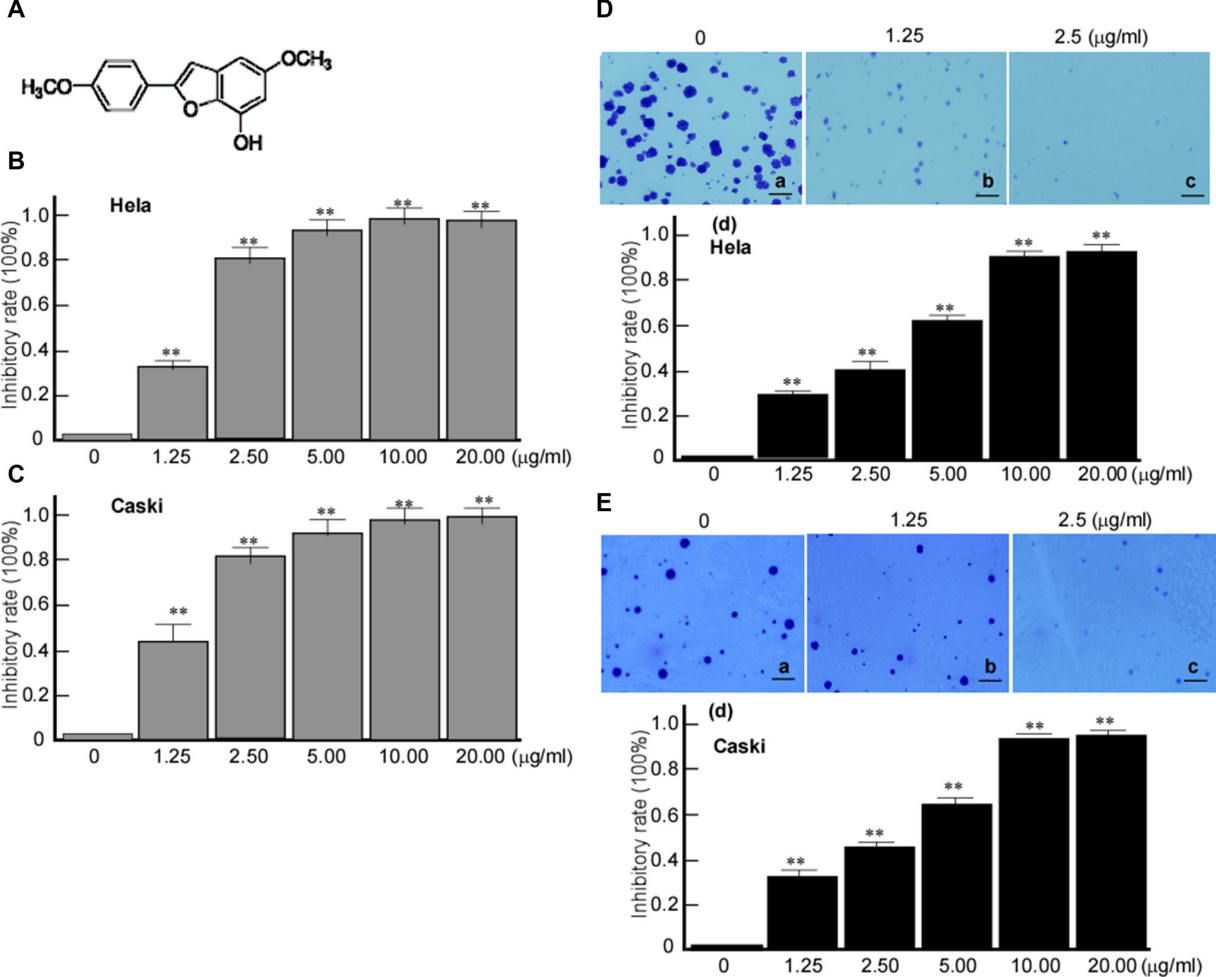
Inhibitory effects of Ary on the growth and colony formation of cervical cancer cells (**A**) Chemical structure of Ary. (**B**) MTT assay of Hela cells treated with Ary at the indicated concentrations after 24 h. (**C**) MTT assay of Caski cells treated with Ary at the indicated concentrations after 24 h. The absorbance ratios to the blank control were calculated in MMT results. Data are shown as the mean ± SD of three independent experiments by analysis of Student's *t* test. *P < 0.05; **P < 0.01. (**D**) Hela cells were treated with Ary at the indicated concentrations, and then cultured in soft agar for 2 weeks. After crystal violet staining, cell colonies were counted. (a), blank; (b), 1.25 μg/mL; (c), 2.5 μg/mL; (d), the inhibitory rates were calculated. (**E**) Caski cells were treated with Ary at the indicated concentrations, and then cultured in soft agar for 2 weeks. After crystal violet staining, cell colonies were counted. (a), blank; (b), 1.25 μg/ mL; (c), 2.5 μg/mL; (d), the inhibitory rates were calculated. The inhibitory rates of colony formation were calculated to the blank control. Data are shown as the mean ± SD of three independent experiments by analysis of Student's *t* test. **P < 0.01.

At first, we detected the inhibitory effect of Ary on cervical cancer cells growth and proliferation. Cervical cancer cell lines, HeLa and Caski cells were treated with Ary, 3-(4,5-dimethylthiazol-2-thiazolyl)-5-(3-carboxymethoxyphenyl)-2-(4-sulfophenyl)-2H-tetrazolium bromide (MTT) assay and Soft agar colony formation assay were used to detect growth and proliferation of the treated cell. MTT assay results showed that both Hela and Caski cell growths were inhibited by Ary treatment (Figure 1B, 1C. *P* < 0.01), displayed a dose dependent manner (Figure 1B, 1C. *P* < 0.01). And soft agar colony formation assay showed that HeLa (Figure 1D–c, b; Figure 1D–d, *P* < 0.01) and Caski's (Figure 1E–c, b; Figure 1E–d, *P* < 0.01) colony formation in the treated groups were significantly low when compared with the control group (Figure 1D–a; Figure 1E–a). Along with increasing Ary's concentration, its inhibitory effect was increased, and the cell colony formation was decreased (Figure 1D–d, *P* < 0.01; Figure 1E–d, *P* < 0.01). The results suggest that Ary could effectively inhibit the growth and proliferation of cervical cancer cell.

**Figure 2:**
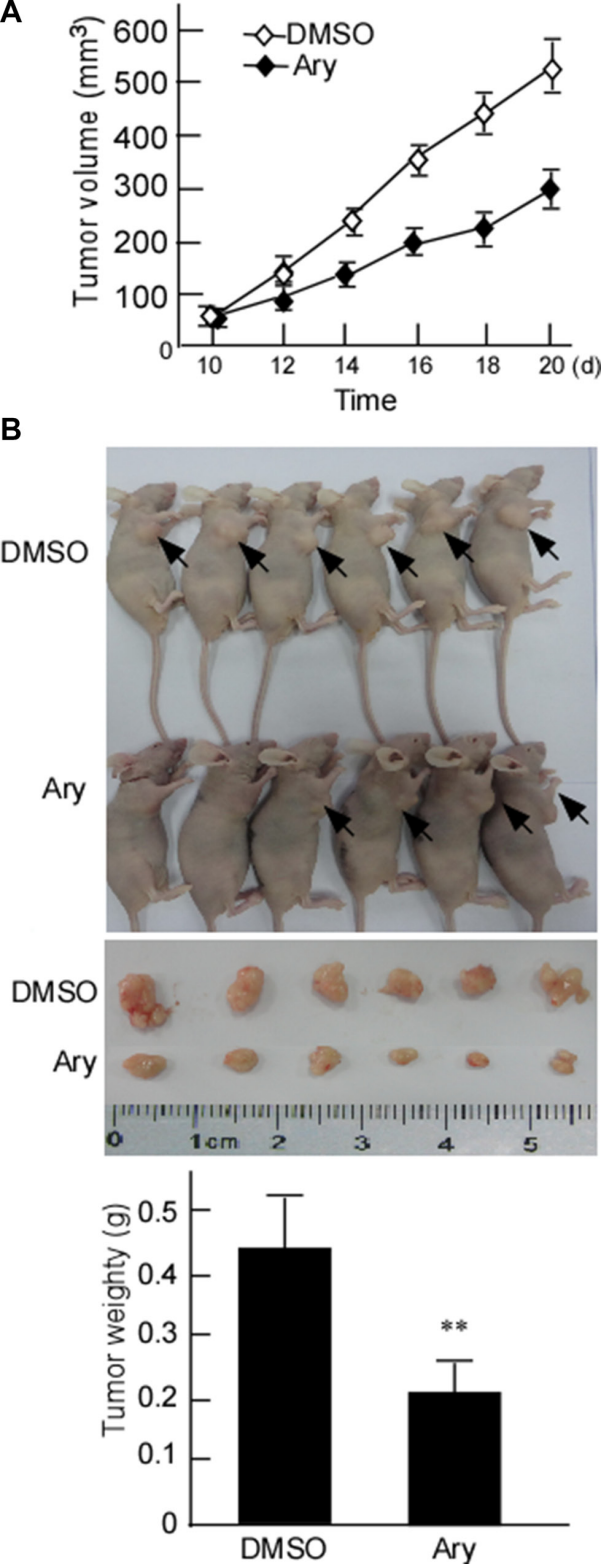
Ary's inhibitory effect on xenograft tumor growth of cervical cancer cells *in vivo* (**A**) The growth curves of the implanted-tumor of cervical cancer. The nude mice were subcutaneously injected with Hela cells. After the nude mice models of cervical cancer cell were constructed, the tumor masses were injected with Ary. The control was injected with DMSO. The tumor volumes were measured. (**B**) The mice were sacrificed after Ary treatment, and the tumors were excised. Tumor weight of nude mice were weighed. Data are shown as the mean ± SD (*n* = 6) by analysis of Student's *t* test. **P < 0.01.

The clearance rate of drug mostly depends on metabolic activity *in vivo* biotransformation process [3, 22]. To further confirm Ary's anticancer effect *in vivo*, Hela cells were transplanted in nude mice. After 10 days, the nude mice models with cervical cancer cells were constructed, and the tumor masses of nude mice were injected with Ary at 50 μg/g (bodyweight) for 10 days, and simultaneously tumor volumes were measured [21]. After treatment 12 days, the implanted tumors were significantly small when compared with the control groups (Figure 2A). After treatment 20 days, the mice were sacrificed, the tumor mass was peeled, its masses were weighed. The tumor weights in Ary group were significantly light when compared with the control groups (Figure 2B–a, b, *P* < 0.01). The results indicated that Ary can inhibit cervical cancer growth *in vivo*.

### Ary induces cervical cancer cells apoptosis through mitochondrial

**Figure 3:**
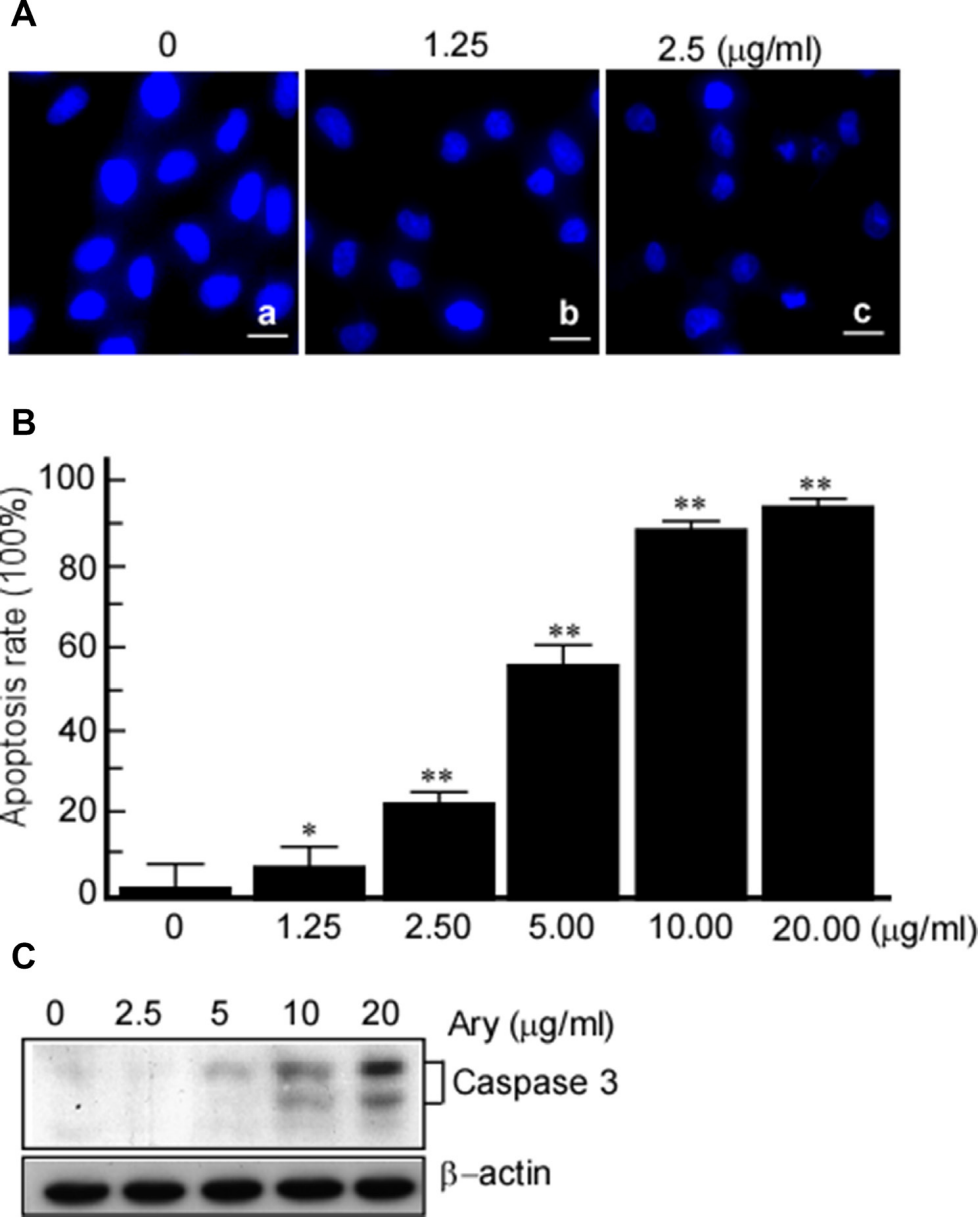
Ary induces cervical carcinoma cell apoptosis (**A**) Hela cells were treated with Ary at the indicated concentrations for 24 h, and then stained with DAPI. The changes of nuclear morphology were observed under a fluorescence microscope (400 ×). (**B**) The treated cells were stained with Annexin V-PI, apoptosis cells were counted using flow cytometry, and the cells apoptosis rates were calculated. *P < 0.05; **P < 0.01. (**C**) Caspase 3 was detected in the treated cells with western-blotting.

In this step, we also observed whether Ary induces cervical cancer cells apoptosis. After Hela cells were treated with Ary, the treated cells were stained with DAPI. The changes of nuclear morphology were observed under fluorescence microscope. The results showed that after Ary treatment with 1.25 μg/mL, the cell nucleus became irregular and small, and cytoplasm was concentrated and marginalized (Figure 3A–b), the treated cells had typical apoptotic bodies when Ary concentration increased to 5 μg/mL (Figure 3A–c), however, in the control group, the cell nucleus were round and color uniformity (Figure 3A–a). The treated cells were stained with Annexin V-PI, apoptosis cells were counted using flow cytometry. The apoptosis rates increased when Ary concentration increase (Figure 3B). Thereafter, the caspase 3 was detected in Hela cells with Ary treatment. The results showed that caspase 3 was activated when Ary treatment, and caspase 3 increased with Ary concentrations increase (Figure 3C).

**Figure 4:**
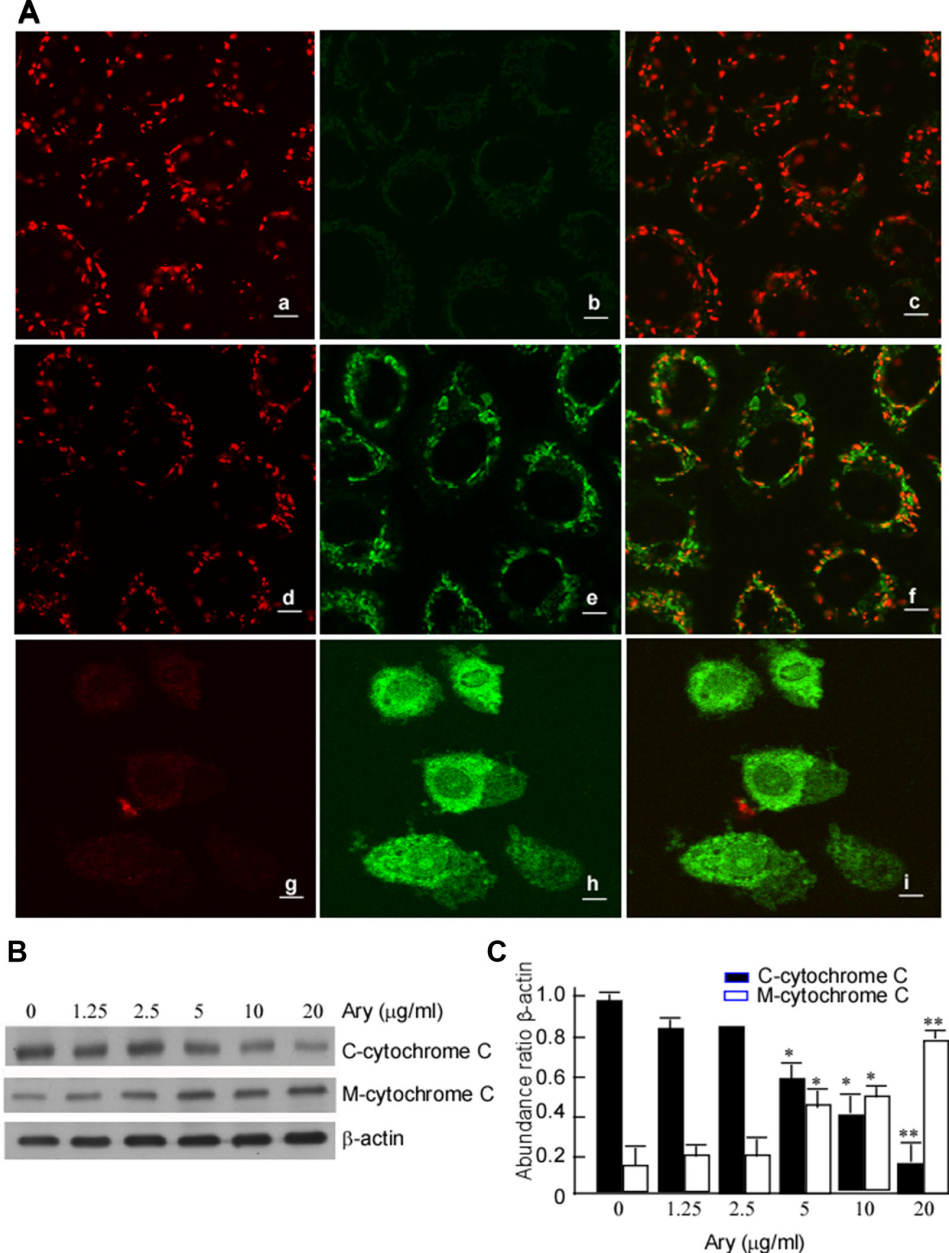
Ary induces the changes of mitochondrial membrane potential (**A**) Hela cells were treated with Ary at the indicated concentrations for 24 h. The treated cells were stained with J-aggregates (red fluorescent) (a, d, g) and JC-1 monomers (green fluorescent) (b, e, h), and observed with confocal microscopy. (a) and (b), blank; (c), emerge of a and b; (d) and (e), 1.25 μg/mL; (f), emerge of d and e; (g) and (h), 2.5 μg/mL; (i), emerge of g and h. (**B**) C-cytochrome C and M cytochrome C expressions were detected in Hela cells treated with Ary at the indicated concentration using Western-blotting. (**C**) The abundance ratios of C-cytochrome C or M-cytochrome C expressions to β-actin were calculated. The data were shown as the mean ± SD of three independent experiments by analysis of Student's *t* test. *P < 0.05, **P < 0.01, *vs* 0 μg/mL.

Decreased mitochondrial membrane potential (MMP) is an early sign of apoptosis event. To further probe the mechanism of Ary-inducing cervical cancer cell apoptosis, Confocal microscopy was used to observe MMP in the treated cell. The results showed that Ary treatment group at 5 μg/mL had a weak staining of J-aggregates (red fluorescent) (Figure 4A–d) and strong staining of JC-1 monomers (green fluorescent) (Figure 4A–e), when Ary concentration was increased to 10 μg/mL, the red fluorescence completely disappeared (Figure 4A–g), only had a strong green fluorescence (Figure 4A–e). However, the control group had strong staining of J-aggregates (Figure 4A–a) and weak staining of JC-1 monomers (Figure 4A–b). These suggest that Ary may induce cervical cancer cell MMP change. Cytochrome C is an important protein which participates in the electronic respiratory chain proteins of mitochondria, it is released into cytoplasm from mitochondria When cells apoptosis [19]. In the next step, we also observed cytochrome C changes in the cells with Ary treatment. The results showed that cytochrome C was increased in cytoplasm, but reduced in mitochondria in the treated cells, displaying a dose-dependent manner (Figure 4B, 4C). These results showed that Ary may induce cervical cancer cell apoptosis through mitochondria degradation.

### Ary arrests cervical cancer cell cycle at G1/S-phase through Cyclin A and Cdk2

**Figure 5:**
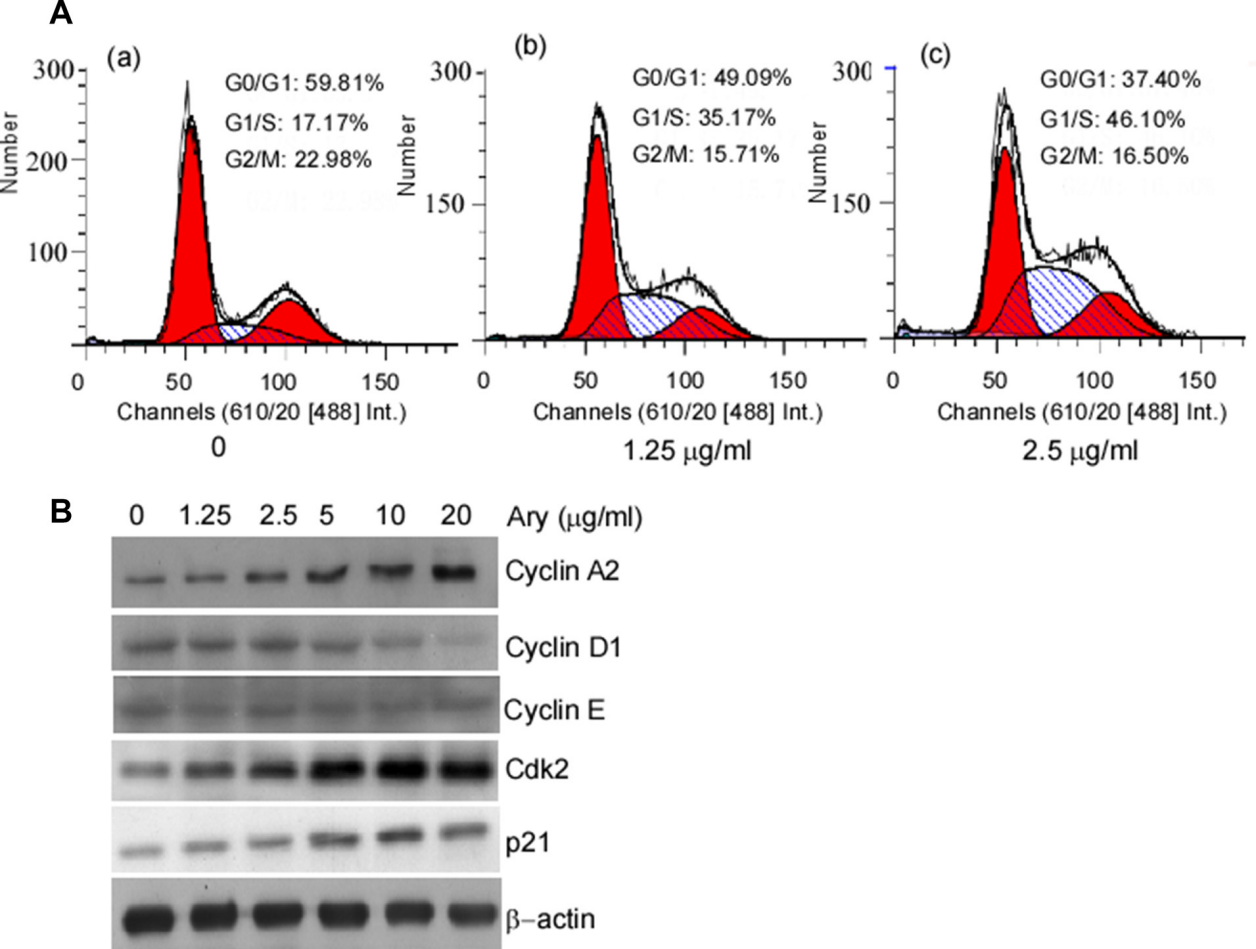
Cell cycle distribution of the cells with Ary treatment (**A**) Hela cells were treated with Ary at the indicated concentration for 24 h. Cell cycle distributions of the treated cells were analyzed by flow cytometry. (a), the blank; (b), 1.25 μg/mL; (c), 2.5 μg/mL. (**B**) Cyclin A2, cyclin D1, cyclin E, Cdk2 and p21 expressions were detected in the treated cells using Western-blotting.

**Figure 6:**
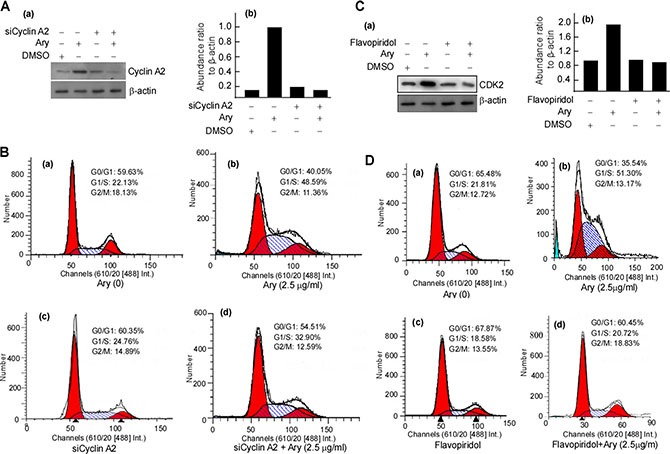
Ary induces cervical carcinoma cell arrest at G1/S through cyclin A2 and Cdk2 (**A)** HeLa cells were transfected with siCyclinA2 for 24 h, and then were treated with Ary at 2.5 μg/mL for 24 h. Cyclin A2 was detected in the treated cells with Western-blotting (a), and the abundance ratios of cyclin A2 expressions to β-actin were calculated (b). (**B**) Cell cycle of the treated cells was analysed by flow cytometry. (a), blank; (b), 2.5 μg/mL; (c), siCyclinA2; (d), siCyclinA2 and Ary. (**C**) HeLa cells were pretreated with flavopiridol for 24 h, and then were treated with Ary at 2.5 μg/mL for 24 h. Cdk2 was detected in the treated cells with Western-blotting (a), and the abundance ratios of Cdk2 expressions to β-actin were calculated (b). (**D**) The treated cells were analyzed by flow cytometry. (a), blank; (b), 2.5 μg/mL; (c), flavopiridol; (d), flavopiridol and Ary.

The previous results showed that Ary inhibits cell growth. We continue to verify whether Ary affects cell cycle progression. HeLa cells were treated with Ary at 1.25 μg/mL or 2.5 μg/mL for 24 h, and then their cell cycle was analyzed using flow cytometry assay. Compared with the control group (17.17%), the treated cells at both Ary 1.25 μg/mL and 2.5 μg/mL had a high percentage of G1/S-phase, 35.17% and 46.10% respectively. G1/ S- phase cell percentage was higher in 2.5 μg/mL than that in 1.25 μg/mL (Figure 5A–b, c). These indicated that HeLa cells were suppressed in G1/S-phase by Ary. As we all know, cyclin A2 and Cdk2 have an important role in the G1/S- phase. And then cyclin A2, cyclin D1, cyclin E, Cdk2, and p21 expressions were detected. The results showed that after Ary treatment, cyclin A2, Cdk2 and p21 expressions were increased, and cyclin D1 decreased, displaying a dose-dependent manner (Figure 5B). To further investigate the function of cyclin A2 in Ary-mediated G1/S arrest, siRNA-cyclinA2 (siCylinA2) was used to knockdown cyclin A2 expression. Western-blotting analysis showed that siCyclinA2 effectively inhibited Ary-mediated cyclin A2 expression (Figure 6A–a, lane 4 *vs* 2 in upper panel; Figure 6A–b, lane 4 *vs* 2). The flow cytometry assay showed that the proportion of G1/S-phase was significantly increased when Ary treatment (Figure 6B–b), this increase was blocked by siCyclinA2 (Figure 6B–d). These suggest that cyclinA2 plays an important role in Ary-induce G1/S arrest. Furthermore, to probe Cdk2's roles in Ary-mediated G1/S arrest, Cdk2 inhibitor, Flavopiridol was used to inhibit Cdk2 expression, and then cell cycle of the treated cell was analyzed. The results showed that Flavopiridol effectively inhibited Cdk2 expression (Figure 6C–a, b), similarly, Ary-induced G1/S arrest was significantly decreased when Flavopiridol treatment (Figure 6D–d). The results demonstrated that Cdk2 may also plays some role in Ary-induce G1/S arrest.

### Ary induces G1/S arrest through sustained activation of ERK1/2

**Figure 7:**
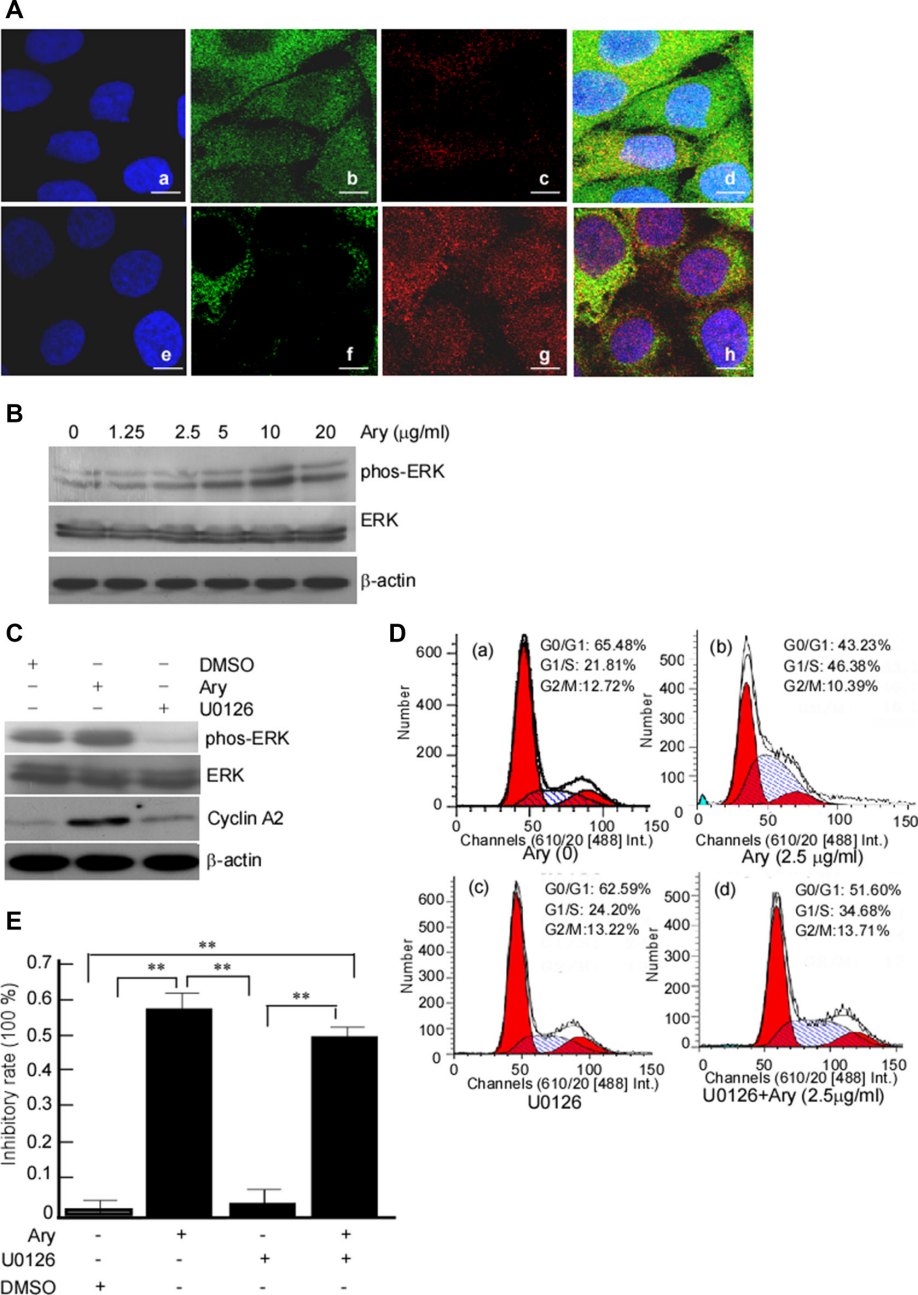
Ary-induced cell cycle arrest at G1/S phase through upregulating ERK1/2 phosphorylation (**A**) Hela cells were treated with Ary at the indicated concentration for 24 h, and phos-ERK1/2 and ERK1/2 were detected in the treated cells using Western-blotting. (**B**) The untreated cells (a, b, c, d) and the treated cells (e, f, g, h) were stained with ERK1/2 mouse mAb (green), phosphorylation of ERK1/2 rabbit mAb (red) and DAPI, respectively. ERK1/2 and phos-ERK1/2 in treated cells were observed under Confocal microscopy. (a, e), DAPI; (b, f), ERK1/2; (c, g), phos- ERK1/2. (**C**) Hela cells were pretreated with U0126 at 10 μM for 2 h, then treated with 2.5 μg/ mL Ary treatment for 24 h. The expression ERK1/2, phos-ERK1/2 and CyclinA2 proteins were detected using Western-blotting. (**D**) The treated cells were analyzed by flow cytometry. (a), blank; (b), 2.5 μg/mL; (c), U0126; (d), U0126 and Ary. (**E**) Colony formation ability of the treated cells was determined using soft agar colony formation assay, and the inhibition rates of colony formation were calculated. Data were shown as the mean ± SD of three independent experiments by analysis of Student's *t* test. *P < 0.05, **P < 0.01.

Mitogen-ativated protein kinase kinase (MAPKK) is involved in many cellular biological functions including proliferation, differentiation, motility and death. ERKs (ERK1/2) are main members of MAPKKs pathway [23, 24]. ERK1/2 is activated through phosphorylation of activation-loop residues threonine (Thr) 202/tyrosine (Tyr) 204 and Thr 185/Tyr 187, respectively. Activated ERK1/2 translocates into nucleus and participates in the regulation of G1- to S-phase transition, and the nuclear translocation of ERK1/2 is served as an anticancer target [25, 26]. So, we want to clarify whether ERK-pathway was activated in G1/S-phase arrest induced by Ary. The results showed that phos-ERK1/2 was increased after Ary treatment, displaying a dose-dependent manner (Figure 7A, upper panel), while total ERK expression did not change (Figure 7A, meddle panel). Further, ERK nuclear translocation was observed. The immunofluorescence assay results showed that after Ary treatment, phos-ERK1/2 in the cytoplasm was decreased when compared with the control (Figure 7B, f *vs* b), while it was increased in the nucleolus (Figure 7B, g *vs* c). This indicated that phos-ERK1/2 was induced from cytoplasm to nucleolus by Ary treatment. To further investigate phos-ERK1/2's role in Ary-mediated G1/S-phase, an inhibitor of phos-ERK1/2, U0126 was used to inhibit phos-ERK1/2 expression. After the cells were treated with U0126, ERK phosphorylation was blocked (Figure 7C, lane 3 in upper panel) and CyclinA2 expression was also reduced (Figure 7C, lane 3 in middle panel), simultaneously the ratios of G1/S-phase were reduced compared with the control group without U0126 (Figure 7D, d *vs* b, *P* < 0.05). Furthermore, we also analyzed the inhibition of Ary on cell soft agar colony formation when U0126 inhibited ERK phosphorylation. Compared with the group, the inhibition rates were reduced (Figure 7E, lane 4 *vs* 2, *P* < 0.05). Over all, these results suggest that Ary may activate phosphorylation ERK1/2 pathway to arrest cervical cancer cell in G1/S-phase and block the cell proliferation.

## DISCUSSION

Ary is a novel natural product which is isolated from *Livistona*, several studies have documented the anti-cancer activity of Ary [2, 20, 27]. But it has rare reports about Ary's effect on cervical cancer and its mechanism. In this study, we showed that Ary can effectively inhibit the proliferation and colony growth of HeLa and CaSki cells *in vitro*, and it can also inhibit cervical cancer xenograft growth *in vivo*, this indicated that Ary has some therapeutic effect on cervical cancer.

To further clarify its mechanism, we detected Ary's effect on cervical cancer cell apoptosis and cell cycle. It is found that Ary can indeed induce cervical cancer apoptosis, which is supported by evidences including morphology change and caspase 3 activation in Ary treated cells. Generally, apoptosis is a type of cell self-destructive in an orderly way by a series of signal cascades which include many gene products and cytokines. Caspase-3 activation plays an important role in the development of apoptosis [28, 29]. In the Ary treated cells, we observed apoptosis morphology changes, and detected the changes of caspase 3. In the further study on mechanism, we found that Ary decreased J-aggregates and increased JC-1 monomers, additionally, Ary also increased cytoplasm cytochrome C and decreased mitochondria cytochrome C. We think that Ary induces cell apoptosis through mitochondria.

The Ary-treated cells were used to analyze cell cycle, we found that Ary can arrest cervical cancer in G1/S phase. Cyclin A2 is a cell cycle protein which predominantly expresses in S phase, simultaneously activates the partner of Cdk2 to regulate the initiation and progression of DNA synthesis [30, 31]. Cdk2 is a member of the Cyclin Dependent Kinase (Cdk) family, which is associated with cyclin A or cyclinE. Cdk inhibitor and p21Cip1 restricts cell to G1/S phase and apoptosis [32–36]. This study demonstrated that Ary can restrict cervical cancer cells in G1/S phase along with cyclin A2 and Cdk2 expression increase. When cyclin A2 gene expression was blocked with siRNA and Cdk2 was inhibited with Flavopiridol, Ary-induced G1/S phase arrest was dramatically decreased. This suggests that cyclin A2/Cdk2-associated kinase activation may be responsible for Ary-mediated G1/S phase arrest.

ERK is extracellular regulated protein kinase which involves in many cellular programs. Phosphorylation activation of ERK translocates from the cytoplasm to the nucleus, and participates in the biological response of cells [37, 38]. Some studies suggest that the regulation of ERK1/2 in G1- to S-phase transition and the nuclear translocation of ERK1/2 served as an anticancer target [23, 24, 39]. However, in this study, we found that Ary induced ERK phosphorylation, and the phosphorylation levels were positively correlated with Ary doses, when ERK1/2 phosphorylation was inhibited, the ratio of G1/S-phase was reduced. Yang TY, et al. found that pemetrexed mediated S-phase arrest and apoptosis conjugated with sustained activation of ERK and Cdk2/Cyclin A, and further verified that the sustained activation of ERK results in S-phase arrest [40]. ERK is thought to be a downstream component of an evolutionarily conserved signaling module that is activated by the Raf serine/threonine kinases, Raf activates the MAPK/ERK kinase (MEK)1/2 dual-specificity protein kinases, which then activate ERK1/2 [41]. Based on these, we speculate that MAPK/ERK kinase (MEK)1/2 phosphorylating ERK is involved in Ary anti-cervical cancer, MEK1/2 may be its upstream molecular. Ary may constantly activate the phosphorylation ERK1/2 to locate the nucleolus and to delay the cervical cancer cells in G1/S-phase to prevent them proliferation.

It is documented that HPV is associated with cervical cancer, and it play an important role in cervical cancer pathogenesis. Whether Ary's anti-cervical cancer is associated with HPV infection? Based on our previous works, Ary has effects on non-cervical cancer which is HPV negative [20, 21]. We speculate that Ary has anticancer effect not only to the cervical cancer with integrated HPV genomes, but also to other cancers with HPV-negative. We think that Ary's anti-cancer effect should not be linked with HPV. The studies *in vitro* showed that Ary could significantly inhibit the growth, proliferation and colony formation of cervical cancer cells. However, most of drug's concentration and clearance rate depends on drug metabolism activity *in vivo* through biotransformation, the effect of drug *in vivo* should be exactly therapeutic effect [39, 42]. In this study, to confirm Ary's effect on cervical cancer *in vivo*, we established a human cervical cancer xenograft models, and validate its therapeutic effect. Ary significantly inhibited the growth of cervical cancer implanted-tumor. We speculate that Ary will be a novel curative medicine, and this will provide a new therapeutic strategy for cervical cancer.

**Figure 8:**
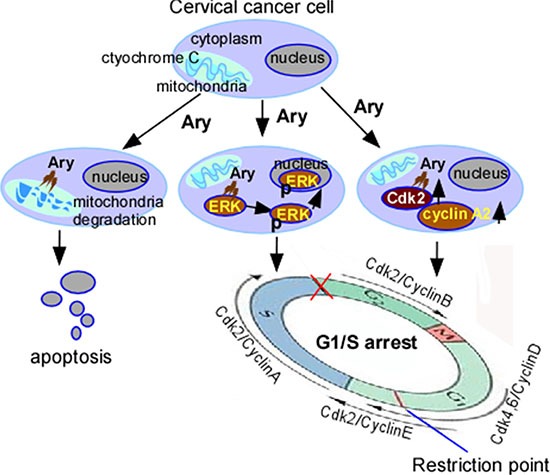
Schematic illustration of Ary-induced anticancer effect on cervical cancer Ary induces cervical cancer cell apoptosis through mitochondrial. Ary induces cell G1/S phase arrest though increasing phos-ERK1/2, cyclin A2, Cdk2 expression and phos-ERK1/2 nuclear translocation.

In summary, our data showed that a novel natural product, Ary can effectively treat cervical cancer *in vivo* and *in vitro*, its mechanism is that it induces cervical cancer cell apoptosis through mitochondrial membrane potential, and arrests cells at G1/S-phase through constantly activating ERK1/2 to locate the nucleolus and upregulating cyclin A2 and Cdk2 expression (Figure 8). In addition, our study also suggested that the sustained-phosphorylation ERK and up-regulation cyclin A2 and Cdk2 elicit cell G1/S arrest.

## MATERIALS AND METHODS

### Reagents and antibodies

Ary was extracted and isolated from the fruits of *L. chinensis* by Hubei University of Traditional Chinese Medicine (Wuhan, China), its purity is 92%. Its chemical structure is shown at Figure 1A. Ary was dissolved in dimethyl sulphoxide (DMSO) for storage fluid, the storage fluid was diluted using cell culture medium for the indicated concentration. Its stability is 98.3% in DMSO, and 97.4% in cell culture medium (shown at Supplementary Table S1). MTT, DAPI, Annexin V, fluorescein isothiocyanate (FITC) reagents were purchased from Invitrogen Corporation (CA, USA). Antibodies against cyclin A2, cyclin D1, cyclin E, Cdk2 or p21 were purchased from Abcam Company (MA, USA). Antibodies to ERK1/2 or phos-ERK1/2 (Thr202/Tyr204) were obtained from Cell Signaling Technology Company (Danvers, MA, USA). Antibody against β-actin, normal mouse IgG, sodium dodecyl sulfate (SDS) lysis buffer, phenylmethane-sulfonyl fluoride (PMSF) and BCA Protein Assay kit were purchased from Santa Cruz Biotechnology (CA, USA). The inhibitors of U0126 (an inhibitor for ERK1/2), Flavopiridol (an inhibitor for Cdk2) were purchased from Sigma company (MO, USA). DMSO, Triton X-100, horseradish peroxidase-linked anti-mouse immunoglobulin G, anti-rabbit immunoglobulin G, antibody against cytochrome C or caspase 3, and Mitochondrial Membrane Potential Assay kit with JC-1 were purchased from Biyuntian Company (Shanghai, China). Mitochondria Isolation Kit was purchased from Pierce Biotechnology (MA, USA). Double-Stranded cDNA Synthesis Kit and Lipofectamine™2000 were purchased from Invitrogen Company.

### Cell line and cell culture

Human cervical cancer cell lines, Hela (HPV18-positive) and Caski cell lines (HPV16-positive) were obtained from American Type Culture Collection (ATCC, ML, USA). Hela and Caski cells were cultured in Dulbecco's Modified Eagle's Medium (DMEM) (Gibco, YK, USA) which supplemented with 10% fetal bovine serum (FBS) (Hyclone, UT, USA) and 1% antibiotics (100 U/mL penicillin,100 μg/mL streptomycin), and incubated in a humidified atmosphere with 5% CO_2_ at 37°C.

### MTT assay

MTT assay was performed as previously described [43]. Briefly, HeLa and Caski cells were seeded in 96-well plate at a density of 5×10^3^ cells/well, and incubated for 24 h. The cells were treated with Ary at 1.25, 2.5 l, 5, 10 and 20 μg/mL, the saline was served as a blank control. After being incubated for 24 h, the cells were changed its media, and incubated again with 20 μL of MTT (5 mg/mL)/well for 4 h. The cells' supernatants were discarded, each cell well was added 150 μL DMSO, and then shocked 10 min to dissolve the crystal violet. Finally, the absorbance of cell wells was measured at 490 nm. The inhibitory rate of various concentrations was calculated, inhibition rate = 1–OD value of experimental group/control group OD value × 100%.

### Soft agar colony formation assay

Cell colony formation assay was performed as previously described [44]. Briefly, the treated cells at 1 × 10^3^ cells/well were suspended in 1 mL of DMEM containing 0.6% low-melting-point agarose (Amresco, USA) and 10% FBS, and plated on a bottom layer containing 0.3% agarose and 10% FBS in 6-well plate in triplicate. The 6-well cell plates were added Ary at 1.25, 2.5, 5, 10 or 20 μg/ mL. After being incubated for two weeks, the cell plates were stained with 0.1% crystal violet for 15 min, and the colonies were counted under a microscopy.

### Cervical cancer cell xenograft experiments

Animal experiments were performed as previously described [44]. Briefly, 20 female BALB/c nude mice (approximately four to six weeks old) were purchased from Guangdong Province Medical Animal Center (Guangzhou Guangdong), fed in a specific pathogen-free environment. After one week, the mice were injected subcutaneously with Hela cell (5 × 10^7^) or Caski cells (5 × 10^7^). The injected mice were randomly divided into two groups (*n* = 6) when the tumors reached a same palpable size. One group was injected with DMSO solvent, served as the control group. Another group received Ary treatment at a dosage of 50 μg/g (bodyweight) for 10 days [21]. The mice tumor block length diameter L and short diameter D were measured with a vernier caliper at 1 time per 2 days, and tumor volume was calculated according to the formula: V = L × D^2^ /2 (18). Mice were sacrificed after 20 days or while the mice appeared moribund, tumors were excised carefully and weighed tumors weight.

### Morphology detection of cell apoptosis

The cells were seeded in 6-well plate, cultured for 24 h, and treated with Ary at 1.25 and 2.5 μg/mL for 24 h. The treated cells were fixed with 4% paraformaldehyde for 15 min, incubated with 0.1% Triton X-100 for 5 min. The fixed cells were stained with DAPI staining working solution at room temperature for 10 min. After being washed with PBS, the cell nuclear morphology was observed under fluorescence microscope.

### Detection of mitochondrial membrane potential

The cells were incubated in a 6-well plate for 24 h, and then treated with Ary at 5 and 10 μg/mL. After being treated for 24 h, the cell medium was removed. After being washed with phosphate-buffered saline (PBS), the cells were added Mito-Tracker Green staining fluid, and then incubated in 37°C for 30 min. After being removed the staining fluid, the cells were washed with PBS, added pre-warmed and fresh cell culture medium. The cells were observed under fluorescence microscopy or Confocal microscope.

### Mitochondria isolation

The treated cells (5 × 10^7^) were collected, and washed with PBS. After being washed, the cells were added 800 μL Mitochondria Isolation Reagent A, and vortexed at medium speed for 5 s, and then incubated tube on ice for 2 min. After ice incubation, the cell suspensions were added 10 μL reagent B, incubated tube on ice for 5 min, and vortexed at maximum speed every minute. After being vortexed, the cell suspensions were added 800 μL reagent C, inverted tube several times to mix, and centrifuged at 700 × g for 10 min at 4°C. The supernatants were collected, centrifuged at 3000 × g for 15 min. After being centrifuged, 500 μL reagent C was added to suspend the pellets, the suspensions were centrifuged at 12,000 × g for 5 min at 4°C. Discarding the supernatant, the pellets were collected, and stored into 4°C for the next experiments.

### Preparation of siRNA and transfection

CyclinA2-siRNA (siCyclinA2) and nonspecific siRNA (simock) were synthesized from Shanghai Gene Pharma Technology Co. Ltd (Shanghai, China). siCyclinA2 sequence is as follows: sense 5′-*CAGGACCAG* GAGAAUAU CATT-3′; antisense 5′-*UGAUAUUCUCCUGGUCCUGTT*-3′. Simock: sense *5*′*-UUCUC CGAACGUGUCACGUTT-3*′, antisense *5*′*-ACGUGACACGUUCGGAGATT-3*′. The siRNAs and Lipofectamine™2000 with Opti-MEM culture medium were mixed gently, then incubated at room temperature for 20 min. The complex was added to each cell wells. The culture medium was exchanged with fresh medium after 6 h, and the cells were collected for flow cytometry analysis after 24 h.

### Annexin V-PI apoptosis rate analysis

The cells were treated with Ary at 1.25, 2.5, 5, 10, and 20 μg/mL for 24 h, collected in tube (1 × 10^6^ cells/ mL), and added 195 μL Annexin V-FITC binding buffer and 5 μL Annexin V-FITC. The cell suspensions were incubated at room temperature in dark for 10 min, and centrifuged. The cell pellets were collected, and added 190 μL Annexin V-FITC binding buffer and 10 μL PI staining solution. After being mixed gently, the cell suspensions were placed in the dark drawer. After being filtrated with 300 mesh nylon net, the cells were immediately detected with flow cytometry.

### Immunofluorescence staining

Immunofluorescence staining was performed as previously described [44]. Briefly, the treated cells were fixed with 4% paraformaldehyde for 15 min, and then added 0.1% Triton X-100 incubation for 5 min. The cells were blocked with 3% bovine serum albumin (BSA), and followed by incubation with mouse antibody against ERK1/2 or Phos-ERK1/2 at 4°C over nigh. The horseradish peroxidase-linked anti-mouse antibodies were used to stain ERK1/2 or phos-ERK1/2. DAPI was use to stain cell nuclear. The cells were observed under Olympus Confocal laser scanning microscope. The cell images were treated using Meta Morph software.

### Western-blotting analysis

Western-blotting analysis was performed as previously described [45]. Briefly, the treated cells (1 × 10^6^) were lysed with 200 μL SDS buffer [50 mM Tris (pH 8.1), 1% SDS, sodium pyrophosphate, β-glycerophosphate, sodium orthovanadate and sodium fluoride, ethylene diamine tetraacetic acid (EDTA), leupeptin, and 1 mM PMSF]. The protein concentration in lysates was measured with BCA Protein Assay kit. 40 μg cell lysates were denatured in 5 × sample loading buffer by heating at 95°C for 10 min. The denatured samples were then separated by 10% polyacrylamide gel. The separated proteins were transferred onto a nitrocellulose membrane (Bio-rad). The membranes were subsequently incubated with 5% non-fat milk in Tris-buffered saline containing 0.05% Tween-20 (TBST) for 1 h to block non-specific binding. The protein membrane was incubated with primary antibody at 4°C over nigh. After being washed, it was incubated with horseradish peroxidase (HRP)-conjugated secondary antibody. Detection was performed by using a chemiluminescent ECL Advance Western blotting detection kit. The primary antibodies used were anti-cyclin A2, anti-cyclin E, anti-Cdk2, anti-cyclin D1, anti-p21, anti-ERK1/2, anti-phos-ERK1/2 (Thr202/Tyr204), or anti-caspase3 antibody. β-actin served as control.

### Flow cytometric analysis

The treated cells were harvested and fixed with cold 70% ethanol. RNA enzyme at 50 μg/mL was added to the cells, and incubated in 37°C for 30 min. The cells were stained with propidium iodide and analyzed its cell cycle by flow cytometry.

### Statistical analysis

All experiments were repeated at least three times and the data were presented as the mean ± SD. Differences between data groups were assessed of Student's *t*-test or analysis of variance (ANOVA) or independent sample *t* test (SPSS16.0). *P* values less than 0.05 was considered statistical significance.

## SUPPLEMENTARY MATERIALS



## Abbreviations

ATCC: American Type Culture Collection
Cdk2: cyclin dependent kinase 2
DMEM: Dulbecco's Modified Eagle's Medium
DMSO: dimethyl sulphoxide
EDTA: ethylene diamine tetraacetic acid
ERK: extracellular signal-regulated kinase
FBS: fetal bovine serum
FITC: fluorescein isothiocyanate
HPV: papillomavirus
HR-HPV: high risk HPV
MAPKK: mitogen-ativated protein kinase kinase
MMP: mitochondrial membrane potential
MTT: 3-(45-dimethylthiazol-2-thiazolyl)-5-(3-carboxym-ethoxyphenyl)-2-(4-sulfophenyl)-2H-tetrazolium bromide
PBS: phosphate-buffered saline
phos-ERK1/2: phosphorylated-ERK1/2
PMSF: phenylmethane sulfonyl fluoride
SDS: sodium dodecyl sulfate
siCylinA2: siRNA-cyclinA2
TBST: tris-buffered saline Tween-20
Thr: threonine
Tyr: tyrosine.
