# Supplementary material for: A novel arylbenzofuran induces cervical cancer cell apoptosis and G1/S arrest through ERK-mediated Cdk2/cyclin-A signaling pathway
Source: Oncotarget. 2016 May 31;7(27):41843–56. doi: 10.18632/oncotarget.9731 (PMC5173100; doi:10.18632/oncotarget.9731)
Supplement: Supplementary file 1 [file oncotarget-07-41843-s001.pdf]

A novel arylbenzofuran induces cervical cancer cell apoptosis and G1/S arrest through ERK-mediated Cdk2/cyclin-A signaling pathway

Supplementary Materials

Evaluation of ary stability

To test the stability of Ary at experiments, Ary was dissolved in dimethyl sulphoxide (DMSO) for storage fluid, the storage fluid was diluted using Dulbecco's Modified Eagle's Medium (DMEM) and 0.1% DMSO for the indicated concentration. The solutions were stored at 37°C for 2, 4, 6, 12, 24, and 48 h. At given time points, Ary concentrations were determined in triplicate. Evaluation of Ary stability was based on the comparison of various time points with the freshly prepared one. Percentage difference between the calculated concentrations obtained for the sample under investigation and freshly prepared sample was evaluated.

Supplementary Table S1: The stability of Ary in difference solutions

Groups	Stability (%)						
	0 h	2 h	4 h	6 h	12 h	24 h	48 h
DMSO	100	99.5	99.4	99.8	99.1	99.2	98.3
DMEM	100	99.2	99.5	99.2	99.0	98.7	97.4
